# 亚胺类共价有机骨架材料在样品前处理中的应用

**DOI:** 10.3724/SP.J.1123.2021.04029

**Published:** 2022-02-08

**Authors:** Hongmei YUAN, Zeyi LU, Yuhuang LI, Chengjiang ZHANG, Gongke LI

**Affiliations:** 1.遵义医科大学药学院, 贵州 遵义 563000; 1. School of Pharmacy, Zunyi Medical University, Zunyi 563000, China; 2.中山大学化学学院, 广东 广州 510275; 2. School of Chemistry, Sun Yat-sen University, Guangzhou 510275, China

**Keywords:** 亚胺类, 共价有机骨架, 样品前处理, 综述, imine, covalent organic frameworks, sample pretreatment, review

## Abstract

亚胺类共价有机骨架(I-COFs)是有机单体根据席夫碱(Schiff-base)反应原理缩合形成的一类新型多孔晶体有机材料。I-COFs具有骨架密度低、比表面积大、孔隙率高、单体种类丰富、孔径尺寸可控、结构可功能化、合成方法多样和物化稳定性好等优点。近年来,I-COFs已成为材料科学领域的研究前沿,并广泛用于气体吸附、存储、催化、传感、光电材料等方面。I-COFs材料优异的物理化学性能使其非常适于用作复杂样品中痕量目标物的分离富集介质,其高比表面积、高孔隙率性能赋予了它极高的吸附负载量,这些性能使得目标分析物可被高效富集;通过控制有机单体的链段长度、几何结构、掺杂元素、取代基团等方面精确调控I-COFs的孔洞结构和功能化基团,从而实现目标痕量物质的选择性富集。目前,I-COFs材料在样品前处理领域作为新型萃取介质已引起了极大关注。该文综述了近年来I-COFs材料的主要类型、合成方法及其在固相萃取、磁性固相萃取、分散固相萃取和固相微萃取方面的研究进展,同时展望了I-COFs在样品前处理领域的发展前景。

## Abstract (en)

Imine covalent organic frameworks (I-COFs), including imine-linked COFs and hydrazone-linked COFs, are a new type of crystalline porous organic materials constructed by the condensation of organic monomers by the Schiff-base reaction. Because they are composed of lightweight elements linked by strong covalent bonds, I-COF materials possess the advantages of low skeleton density, large surface area, high porosity, abundant monomer species, controllable pore size, functionalized structure, diverse synthetic methods, excellent adsorption performance, outstanding physical and chemical stabilities, etc. In recent years, interest in the field of I-COFs has increased tremendously because of their exceptional performance and broad applications in gas storage, gas separation, catalysis, sensing, photoelectric materials, sample pretreatment, drug delivery, and other fields. To date, imine bonds are one of the most widely used covalent bonds in COFs, and represent one of the most important ways to obtain I-COFs with excellent chemical stabilities. The synthesis methods for I-COFs include solvothermal synthesis, microwave synthesis, mechanochemical grinding synthesis, and room-temperature synthesis methods. Solvothermal synthesis is the most extensively used method for the production of I-COFs with high specific surface areas and good thermal stabilities. The microwave synthesis method is conducive to the rapid synthesis of COFs in industry, providing a more time-saving, simpler, and safer route for large-scale preparation of I-COFs. The mechanochemical grinding synthesis of porous solids has gained importance as an alternative to conventional solvothermal synthesis, because the process is quick, environment-friendly, and potentially scalable. The room-temperature method is characterized by mild reaction conditions and rapid reactions. It is an energy-saving, economic, safe, and green synthesis method, which has emerged as a hot spot in the preparation of I-COFs in recent years. Research progress over the past years on the application of I-COFs in the field of materials science has undoubtedly established the basis of its application in analytical chemistry. Owing to the excellent physical and chemical properties of I-COF materials, they are suitable for use as separation and enrichment media for trace target compounds in complex samples. The high specific surface area and porosity, extended conjugate network skeleton, and *π*-electron-rich nature of the materials endow it with a high adsorption capacity. These materials are highly enriched in target analytes by *π-π* interactions, acid-base interactions, donor-acceptor interactions, hydrogen bonding, hydrophobic interactions, and other intermolecular interactions. Precise control of the microporous structure of I-COFs was obtained by controlling the chain length, geometric structure, doping elements, and substituent groups of the organic monomers. Selective enrichment of target trace substances was achieved by modifying the groups of I-COFs based on the principle of host guest adaptation, molecular sieving, or microporous filling effect. At present, research on the synthesis of I-COF materials is in the stage of rapid development, and many I-COFs with excellent properties and great application potential have been synthesized, allowing widespread application of I-COFs in sample pretreatment medium. This review summarizes the current state-of-the-art on the main types and synthetic methods of I-COFs, as well as the applications of I-COFs in solid-phase extraction, magnetic solid-phase extraction, dispersive solid-phase extraction, and solid-phase microextraction. The prospects of I-COFs in sample pretreatment are also presented.

共价有机骨架(covalent organic frameworks, COFs)是有机单体通过很强的共价键相互连接而形成的一类新型多孔晶体有机聚合物^[1]^。COFs完全由轻质元素组成,具有骨架密度低、比表面积大、孔隙率高、孔径尺寸可控和结构可功能化等优点,近年来成为材料科学领域的研究前沿,已广泛用于气体吸附、存储、催化、传感、光电材料等方面^[2,3,4]^。目前,根据形成共价键的类型,COFs材料主要分为含硼类、三嗪类和亚胺类等。其中,含硼类COFs由于缺电子硼位点易受亲核试剂(如水分子)的攻击,在水、湿气中的稳定性差,大大限制了它的应用;三嗪类COFs通常需要在极高的温度下长时间反应,其制备条件较为苛刻,合成较为困难,且产物的结晶度和规整性较差,因而不利于COFs材料的大量合成和广泛应用。然而,亚胺类COFs的合成条件简单(在室温条件下即可合成)、合成方法多样和化学稳定性好,能稳定存在于常用有机溶剂、水,甚至酸、碱溶液中^[5]^。因此,亚胺类COFs是目前应用最为广泛的一类COFs材料。根据亚胺类COFs材料的结构特点分析,其超高的比表面积、纳米尺寸孔洞、高孔隙率和优异的物理化学稳定性,使其非常适于用作复杂样品中痕量目标物的分离富集介质。亚胺类COFs材料的高比表面积、高孔隙率性能赋予了它极高的吸附负载量,这些性能使得目标分析物可被高效富集;通过控制有机单体的链段长度、几何结构、掺杂元素、取代基团等方面精确调控亚胺类COFs的孔径结构和功能化基团,从而实现目标痕量物质的选择性富集^[6]^。基于亚胺类COFs材料优异的物理化学性能,目前该材料在样品前处理领域作为新型萃取介质已引起了极大关注。本文综述了近年来亚胺类COFs材料在固相萃取(solid phase extraction, SPE)、磁性固相萃取(magnetic solid phase extraction, MSPE)、分散固相萃取(dispersive solid phase extraction, DSPE)和固相微萃取(solid phase microextraction, SPME)方面的研究进展,同时展望了亚胺类COFs材料在样品前处理领域的发展前景。

## 1 亚胺类COFs的主要类型

亚胺类共价有机骨架(imine covalent organic frameworks, I-COFs)是有机单体根据席夫碱(Schiff-base)反应原理缩合形成的一类新型多孔晶体有机材料,主要包括胺与醛缩合形成的亚胺键(C=N)、酰肼与醛缩合形成的腙键(见图1)。

**图1:**
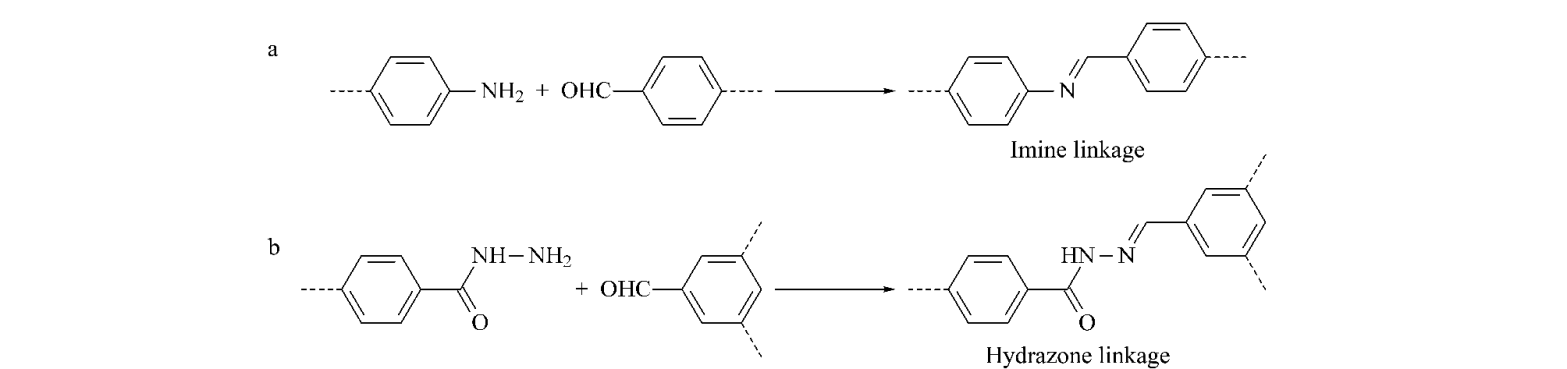
I-COFs材料的类型

### 1.1 亚胺键COFs

伯胺与醛的有机单体通过Schiff-base反应原理缩合形成亚胺键COFs(如图1a)。Uribe-Romo等^[7]^首次采用四(4-氨基苯基)甲烷与对苯二甲醛为有机单体,在溶剂热条件下根据Schiff-base反应得到三维(3D)结构的COF-300, COF-300具有高比表面积和永久开放孔隙,在水和常用有机溶剂中具有良好的化学稳定性。COF-300的合成为新型COFs材料的研制提供了新的思路与方法。随后,基于亚胺键连接的各种二维(2D)和3D新型COFs材料被不断开发出来,并广泛应用于不同领域的研究。如采用1,3,5-三甲酰基苯与对苯二胺为结构单元制备的2D COF-LZU1^[8]^,用四(4-氨基苯基)甲烷和1,3,6,8-四(4-甲酰基苯基)芘为有机单体构建的3D-Py-COF^[9]^。亚胺键COFs由于含有C=N,在酸、碱环境下不稳定,利用COFs骨架中苯环上的烯醇-酮互变异构、形成分子内氢键,可增强COFs材料的稳定性。Kandambeth等^[10]^用1,3,5-三甲酰基间苯三酚(Tp)和对苯二胺(Pa-1)、2,5-二甲基对苯二胺(Pa-2)为有机单体合成了TpPa-1和TpPa-2,两种材料在沸水、酸/碱环境中都具有很好的稳定性;随后,Chandra等^[11]^采用1,3,5-三甲酰基间苯三酚和对苯二胺及其衍生物、联苯二胺(BD)及其衍生物合成了具有优异化学稳定性的一系列TpPa(TpPa-1、TpPa-2、TpPa-NO_2_、TpPa-F_4_)和TpBD(TpBD、TpBD-Me_2_、TpBD-(OMe)_2_、TpBD-(NO_2_)_2_)材料。通过2,5-二羟基对苯二甲醛(Dha)和5,10,15,20-四(4-氨基苯基)卟啉(Tph)缩合形成的DhaTph,由于DhaTph骨架中-OH与邻位的C=N中心能形成分子内氢键,该材料在水、酸中也表现出良好的稳定性^[12]^。采用将甲氧基结合到COFs孔壁中来增强层间相互作用,也是获得稳定亚胺COFs的重要方式,Halder等^[13]^以2,4,6-三甲氧基-1,3,5-三甲酰基苯(TpOMe)和不同结构的芳香二胺为反应单体,对甲苯磺酸为催化剂,在溶剂热条件下快速构建了6种具有超高稳定性的亚胺COFs, TpOMe中的甲氧基与亚胺键间的层间氢键可提供足够的空间位阻和疏水作用,使材料在H_2_SO_4_(18 mol/L)、HCl(12 mol/L)、NaOH(9 mol/L)、沸水和常用有机溶剂中都具有出色的化学稳定性。手性COFs在手性催化、手性分离等领域极具应用价值,但手性COFs的合成目前仍具有很大挑战。Han等^[14]^通过四(4-氨基苯基)甲烷和手性四芳基-1,3-二氧戊环-4,5-二甲醇衍生的四醛进行缩合反应,采用“自下而上”的策略合成了3D手性共价有机骨架(CCOF-5),再通过后合成氧化CCOF-5骨架中的亚胺键,从而转化成稳定性更高的酰胺键CCOF-6,两种材料均可作为HPLC手性固定相分离外消旋的醇类化合物,该研究为3D手性CCOF的制备和应用开辟了新途径。光学纯的1,1'-二-2-萘酚是有机合成和材料科学中最重要的手性源物质,Wu等^[15]^采用6,6'-二氯-2,2'-二乙氧基-1,1'-联二萘-4,4'-二醛与四(4-氨基苯基)乙烯、1,3,5-三(4-氨基-3,5-二异丙基)苯为有机单体,以溶剂热法制备了具有2D层状四方形或六边形的手性荧光COFs(CCOF 7和CCOF 8),由于COF纳米片有较强的荧光性质、特异的分子识别位点和优异的化学稳定性,可实现选择性、高灵敏传感检测对映体分子。多孔材料的微孔结构有利于物质的吸附,介孔结构有利于物质和能量的传递,同时具有微孔和介孔结构的COFs材料能大大改善材料的应用性能。Zhu等^[16]^通过选择1,4'-(二(4-甲酰苯基)氨基)-[1,1-联苯]-3,5'-二醛(BABD)与对苯二胺、联苯二胺为结构单元,利用溶剂热法合成了具有双孔的荧光BABD-Tp-1(原文献写作COF-BABD-DB)和BABD-BD(原文献写作COF-BABD-BZ),杂化孔径的COFs具有光谱和颜色变化特征,对2,4,6-三硝基苯酚表现出极高的选择性和灵敏度。多级孔COFs材料的设计与合成为其应用提供了丰富的选择性。Li等^[17]^采用双官能团(甲酰基和氨基)的1,6-二(4-甲酰基苯基)-3,8-二(4-氨基苯基)芘单体,通过“二合一”自聚缩合形成2D Py-COFs,该材料具有高结晶性、高孔隙率和优异的化学稳定性。“二合一”策略为设计新型有机单体与COFs材料提供了一种全新的思路与方法。随后,Li等^[18]^设计合成了具有对位、间位和邻位取代异构体结构的A_2_B_2_型四苯基苯单体(*p*-、*m*-和*o*-TetPB),通过自聚反应构建了异构体骨架的2D TetPB-COF,该材料能高选择性地吸附维生素B_12_。Nguyen等^[19]^提出了一种基于三角形和正方形连接的fjh拓扑结构通过构象设计构建单元获得选择性形成亚胺共价有机框架的策略,采用1,3,5-三甲基-2,4,6-三(4-甲酰基苯基)苯和1,1,2,2-四(4-氨基苯基)乙烯为结构单体,以溶剂热法合成了3D COF-790,该材料具有永久的孔隙率和高比表面积(2650 m^2^/g),此方案为设计构建新型3D COF提供了新理念。碗形的杯[4]芳烃具有特异的主体-客体化学性质,是一种通用的超分子构建基块。最近,Garai等^[20]^报道了采用醛基功能化的杯[4]芳烃(CX4-CHO)与联苯二胺为结构单元合成了亚胺连接的二维扩展共价有机骨架,通过改变反应混合物的浓度来调节相邻杯芳烃单元之间的相互作用,可选择性形成互穿(CX4-BD-1)和非互穿(CX4-BD-2)框架,结构主链中的碗形杯芳烃部分允许CX4-BD-1中相邻的两层相互交织,使其成为相互贯通的2D层的独特范例。

目前,亚胺键是构筑COFs材料使用最广泛的一种共价键,是获得优异化学稳定性COFs材料最为重要的路径之一。

### 1.2 腙键COFs

酰肼与醛的有机单体反应缩合形成腙键COFs(如图1b)。相对于亚胺键,腙键COFs材料具有更好的物理化学稳定性。Uribe-Romo等^[21]^首次报道了以2,5-二乙氧基对苯二甲酰肼与1,3,5-三甲酰基苯、1,3,5-三(4-甲酰基苯基)苯为有机单体,在溶剂热条件下合成含有腙键的COF-42和COF-43,两种材料具有较高的比表面积和孔隙率,在常用有机溶剂中具有较好的化学稳定性。随后,腙键结构的各种COFs材料相继被报道,Stegbauer等^[22]^以1,3,5-三(4-甲酰基苯基)三嗪(TFPT)和2,5-二乙氧基对苯二甲酰肼为原料合成了当时具有最高比表面积(1603 m^2^/g)的TFPT-COF。COF有序的*π*结构可用于开发发光材料,但是大多数COF的发光强度很弱。Li等^[23]^报道了以1,3,6,8-四(4-甲酰基苯基)芘(TFPPy)和2,5-二乙氧基对苯二甲酰肼(DETHz)为单体研制的TFPPy-DETHz-COF,通过在孔壁上进行精准设计将较少发光的COF转换为发光材料,作为首例阴离子荧光传感能够选择性地检测μg/L的氟离子。Chen等^[24]^采用均苯三甲酰肼(Bth)与2,5-二羟基对苯二甲醛、2,5-二甲氧基对苯二甲醛(Dma)为单体合成了具有功能性O,N,O'螯合位点的Bth-Dha和Bth-Dma。Bth-Dma在固体状态和在水性分散体状态均表现出较强的荧光特征,而Bth-Dha则没有观察到荧光,Bth-Dma作为“关断型”荧光传感器检测水溶液中Fe^3+^,具有出色的选择性和灵敏度。该研究为设计具有功能性结合位点以检测特定金属离子的发光COF传感器提供了新方案。手性COFs具有重要的应用价值,但其合成是一个巨大挑战。Yan等^[25]^从头合成了2D羟基官能化的腙键手性COFs,用对映体2,5-二(2-羟基丙氧基)对苯二酰肼[(*S*)-Hth和(*R*)-Hth]与1,3,5-三甲酰基苯(Bta)为单体构建羟基功能化的手性(*S*)-和(*R*)-HthBta-OH COFs,再用丁二酸酐与对相应的羟基手性COF进行后合成修饰,得到羧基官能化手性(*S*)-和(*R*)-HthBta-COOH COFs,由于原始腙键手性COFs优异的化学稳定性,在经过化学修饰后,羧基官能化的COF保持了同手型和结晶度,且没有发生连接体外消旋和结构塌陷。Qian等^[26]^以2,5-二乙氧基对苯二甲酰肼与1,3,5-三(吡啶醛基)苯为有机单体合成得到腙键结构的COF-DB, COF骨架中含N位点能与不同过渡金属离子发生配位作用,从而提高了材料的结晶度和稳定性,金属化为获得稳定性COF材料提供了新的思路。通常制备COFs材料的有机单体需要刚性芳香环,而采用非刚性单体对于构建新型COFs具有很大的发展潜力。Li等^[27]^报道以柔性烷基胺为结构单元,分子内氢键为网络中的结,通过采用1,3,5-三甲酰基间苯三酚、1,3,5-三甲酰基苯和草酰二酰肼(ODH)作为前体合成了腙键结构的TpODH和BtaODH(原文献写作TFBODH),由于TpODH骨架中能形成不可逆的烯醇-酮互变异构和分子内氢键,从而增强了材料的结晶度和化学稳定性,且具有高比表面积。最近,Bagherian等^[28]^用2,4,6-三(甲酰基苯氧基)-1,3,5-三嗪(TPT)和草酰二酰肼为单体合成了具有大孔尺寸(3.35 nm)和优异化学稳定性的TPT/ODH COF(原文献写作TPT/OH COF)。目前,腙键COFs材料的报道相对较少,解决作为合成前体的酰肼溶解性差的问题,以及发展柔性单体有利于研制出更多结构新型的腙键COFs材料,进一步拓展其应用范围。

由于构建I-COFs的单体含有醛基、氨基等基团,可作为COFs衍生化的位点,实现材料的多样化、功能化。因此,I-COFs是应用最广泛、最具发展潜力的一类新型COFs材料。

## 2 亚胺类COFs的合成方法

COFs是由有机结构基元经热力学控制的可逆聚合而成的多孔晶体,反应溶剂和反应条件是稳定晶态形成的重要影响因素。目前,亚胺类COFs材料的合成方法主要有溶剂热合成法、微波合成法、机械研磨法和室温法。

### 2.1 溶剂热合成法

溶剂热合成法是制备I-COFs材料最常用、最广泛的方法^[4,29,30]^。其制备过程通常是将反应单体和溶剂介质放于耐压耐热玻璃管中,经过液氮冷冻-抽真空-解冻循环2~3次后;密封玻璃管,恒温加热反应一定时间;反应结束后,用合适的溶剂洗脱未反应完全的单体,真空干燥除去客体分子从而获得永久开放的多孔COFs材料。反应溶剂(如溶剂类型、比例)和反应条件(如反应温度、压力、时间等)均是影响晶态形成的重要因素^[17,18,19]^,目前报道的反应溶剂有单一有机溶剂(均三甲苯、1,4-二氧六环、甲醇、乙醇、正丁醇、二甲基乙酰胺、二甲基甲烷、二氯甲烷、甲苯、间甲酚、氯仿、四氢呋喃和乙腈)或混合溶剂(均三甲苯/1,4-二氧六环、1,4-二氧六环/正丁醇、乙醇/均三甲苯、均三甲苯/二甲基乙酰胺、邻二氯苯/正丁醇、二甲基乙酰胺/邻二氯苯、二甲基甲酰胺/水和硝基苯/均三甲苯)。催化剂一般为3 mol/L或6 mol/L的乙酸水溶液;反应压力在0~20 Pa之间;有机溶剂与乙酸水溶液的体积比常用5∶1、10∶1或20∶1;反应温度一般为90~120 ℃;反应时间从2天至9天。与其他制备方法相比,溶剂热合成法研制的I-COFs材料通常具有较高的比表面积,更好的热稳定性,但其反应周期较长、合成条件比较苛刻,成本也较高,限制了I-COFs材料大规模的合成及应用。

### 2.2 微波合成法

与溶剂热法相比,微波合成法具有快速、高效、节能等优点,其反应时间从天到小时,从分钟到秒,将微波法应用于I-COFs的合成,极大地促进了I-COFs材料的发展。Wei等^[31]^首次报道采用1,3,5-三甲酰基间苯三酚和对苯二胺为原料,通过微波加热(100 ℃反应1 h)快速合成了TpPa-COF(MW)。原料在无微波辐射下用传统的溶剂热反应1 h,获得的TpPa-COF(CE)产率仅为8%;与典型溶剂热法合成的TpPa-COF(CS)相比,TpPa-COF(MW)具有优异的结晶度和较高的比表面积(724 m^2^/g),微波合成法大大缩短了反应时间,提高了反应速率。近来,Xu等^[32]^用1,3,5-三甲酰基间苯三酚与2,5-二甲基对苯二胺为原料,在乙酸/均三甲苯/1, 4-二氧六环混合溶剂中,通过微波加热(100 ℃反应1 h)合成了性能最佳的TpPa-2(MW),与机械研磨法制备的TpPa-2(MC)相比,TpPa-2(MW)有更好的晶体结构和更大的比表面积(535 m^2^/g)。Vazquez-Molina等^[33]^报道采用微波快速合成了一系列低聚(乙氧基)侧链功能化的COFs,用1,3,5-三甲酰基间苯三酚分别与不同侧链的2,5-二取代基功能化4,4″-二氨基-对三联苯(TP-R)为有机单体,其中R为H、乙氧基(OEt)、乙二醇单甲醚(OMEG)、二甘醇单甲醚(ODEG)和三甘醇单甲醚(OTEG)。使用250 W微波合成器加热170 ℃反应20 min,在1,2-二氯乙烷/1,4-二氧六环/乙酸的混合溶剂中实现了TpTP-H(原文献写作TfpTP-H)和TpTP-OEt(原文献写作TfpTP-OEt)的合成,而选择正丙醇/邻二氯苯/乙酸为溶剂合成了TpTP-OMEG、TpTP-ODEG、TpTP-OTEG(TfpTP-OMEG、TfpTP-ODEG、TfpTP-OTEG),溶剂类型是制备不同COFs材料的一个重要影响因素。Chen等^[34]^采用1,3,5-三(4-氨基苯基)苯(TAPB)和2,3,5,6-四氟对苯二甲醛(TFA)或对苯二甲醛(PDA)为原料,均三甲苯/乙酸或均三甲苯/1,4-二氧六环为溶剂,在微波功率200 W下加热反应1 h得到TAPB-TFA-COF和TAPB-PDA-COF,其产率达80%~85%。与传统溶剂法相比,微波加热法具有较快的反应速度,但COFs材料的比表面积有所下降。Ding等^[35]^基于微波辅助阴离子快速交换反应合成了新型COFs材料,以溴化乙锭和1,3,5-三甲酰基间苯三酚为原料,1,4-二氧六环/均三甲苯/乙酸为溶剂介质,反应物在玻璃管中经过冷冻-抽真空-解冻循环3次后密封,于微波合成器中100 ℃加热3 h合成了SJTU-COF-Br;随后将SJTU-COF-Br分别分散在氯化钠、醋酸钠或三氟甲磺酸钠的饱和盐溶液中,微波辐射下80 ℃搅拌反应30 min,获得3种新型COFs材料(SJTU-COF-Cl、SJTU-COF-AcO与SJTU-COF-CF_3_SO_3_)。微波合成法缩短了反应时间,快速实现了SJTU-COF-Br与盐溶液中的阴离子交换,基于二氧化碳(CO_2_)和醋酸盐阴离子之间的相互作用,增强了SJTU-COF-AcO对CO_2_的吸附容量。该研究为设计合成新型COFs材料提供了新的方案。Martín-Illán等^[36]^报道了一种以水为溶剂的绿色合成法制备COFs,以1,3,5-三(4-氨基苯基)苯和1,3,5-三甲酰基苯为原料,先将两种反应单体在水溶液中混合,再用乙酸调溶液pH值至2.4。采用200 W微波辐射加热80 ℃反应5 h得到良好结晶度和高比表面的MW TAPB-Bta-COF (原文献写作TAPB-BTCA-COF),其产率(85%)与传统溶剂热反应5天的结果相当。微波合成法有利于COFs材料在工业上进行大量快速的合成,为大规模制备I-COFs材料提供了一种更省时、更简单、更安全的方法。

### 2.3 机械研磨法

机械研磨法具有操作简单、条件温和、方便快捷等特点,在COFs材料的合成应用中具有重要的推动作用。Biswal等^[37]^以1,3,5-三甲酰基间苯三酚和对苯二胺及其衍生物为原料,首次通过室温无溶剂机械研磨法成功制备出TpPa-1(MC)、TpPa-2(MC)和TpBD(MC)。其过程是先将两种反应原料混合放入研钵,然后在室温下研磨,根据不同时间观察到的反应物颜色变化来推测产物的形成过程,45 min后得到深红色的产物。与溶剂热法合成的COFs相比,此法获得的材料比表面积较低,但具有同样的孔道和晶体结构,在强酸、强碱中也具有较好的稳定性,其合成速度是溶剂热的96倍。研磨法为COFs的合成提供了一种新的方法。随后,Das等^[38]^采用液体辅助研磨法(liquid-assisted grinding, LAG),以1,3,5-三甲酰基苯和对苯二胺,1,3,5-三甲酰基间苯三酚和对苯二甲酰肼(Th), 5,10,15,20-四(4-氨基苯基)卟啉与2,5-二羟基对苯二甲醛为原料制备出LZU-1(LAG)、TpTh (LAG)和DhaTph(LAG)。相对于无溶剂研磨法,LAG获得的COFs具有更高的纯度和产率。Wang等^[39]^以1,3,5-三甲酰基间苯三酚与4,4'-偶氮二苯胺(Azo)为单体,采用LAG快速实现了TpAzo材料的制备,具体过程是先将对甲苯磺酸加入研钵,接着加入Azo混合均匀研磨5 min,于研钵中再加入Tp,混合后研磨10 min直到出现颜色变化。然后将水(0.5 mL)逐滴加入混合物中研磨5 min,再将混合物转移至表面皿于马弗炉中170 ℃加热60 s,获得深红色的粉末,最后依次用水、有机溶剂洗涤粉末,真空干燥得到产物。该方法制备的TpAzo材料具有高比表面积、高孔隙率和热稳定性,最后用于SPE应用研究。随后,他们用类似的方法,选择2,6-二氨基蒽醌(DAAQ)与1,3,5-三甲酰基间苯三酚为单体也成功制备出DAAQ-Tp(原文献写作DAAQ-TFP),材料同样具有好的结晶度和高热稳定性^[40]^。Peng等^[41]^以1,3,5-三甲酰基间苯三酚与2,5-二氨基苯磺酸、2,5-二氨基苯-1,4-二磺酸为原料,用等体积的均三甲苯/1,4-二氧六环/乙酸水溶液的混合溶剂(50 μL)辅助反应,在研钵中室温研磨45~60 min,成功制备出NUS-9和NUS-10,其产率分别为80%和76%。研磨法通常获得的I-COFs比表面积较小,但为快速、大量合成I-COFs材料提供了新的思路及可能性。

### 2.4 室温法

室温法具有反应条件温和、反应快速等特点,是一种节能、经济、安全、绿色的合成方法,近年来成为I-COFs材料制备的热点。Yang等^[42]^报道了一种简单、方便的室温溶液合成法制备球形COF,采用1,3,5-三甲酰基间苯三酚和联苯二胺为单体,在室温下30 min内合成球形TpBD,与溶剂热法(120 ℃反应3天)和机械研磨法(45 min)相比,该方法的合成时间最短、操作最简单。TpBD材料在水、有机溶剂、强酸强碱中均具有良好的化学稳定性和高热稳定性,可用于高分辨气相色谱的应用。随后,He等^[43]^采用室温溶液合成法研制出新型花束状磁性TpPa-1,材料独特的花束状结构使其具有高比表面积、高孔隙率和超磁性,作为MSPE吸附剂表现出优异的吸附性能。Lin等^[44]^报道采用1,3,5-三-(4-氨基苯基)苯和对苯二甲醛为单体,四氧化三铁(Fe_3_O_4_)为核,在二甲基亚砜(DMSO)溶剂中室温下快速(5 min)合成了核-壳结构的Fe_3_O_4_@COF。材料具有优异的物理化学稳定性,在生物样品中多肽富集方面展现出良好的应用潜力。Lin等^[45,46]^选择2,5-二羟基对苯二甲醛与1,3,5-三(4-氨基苯基)苯为单体,功能化的Fe_3_O_4_纳米粒子为磁核,将混合物加入三口烧瓶于乙酸溶液中室温搅拌反应3天,研制出磁性TAPB-Dha(原文献写作COF-DtTb),该吸附剂实现了水果和牛奶中的有机磷农药的分离富集。Ji等^[47]^用氰戊菊酯为印迹模板,Sc(OTf)_3_为催化剂,1,3,5-三(4-氨基苯基)苯与1,3,5-三甲酰基间苯三酚为结构单体,在室温下合成了分子印迹共价有机骨架复合材料(MICOFs)。在MICOFs的合成过程,催化剂是实现便捷制备产物的关键因素,使用传统的催化剂(乙酸、三氟乙酸和对甲苯磺酸)需要在大于90 ℃下反应3天,而用Sc(OTf)_3_则室温反应30 min。该研究是快速制备功能化COFs材料的重要途径。采用类似的合成方法,Zhao等^[48]^采用1,3,5-三甲酰基间苯三酚和2,6-二氨基蒽醌为单体,花青素-3-*O*-葡萄糖苷为印迹模板,研制的磁性MCMIPs-DAAQ材料可高效吸附植物样品中的花青素-3-*O*-葡萄糖苷。Gao等^[49]^以1,3,5-三(4-氨基苯基)苯和2,3,5,6-四氟对苯二甲醛为原料,采用室温溶液合成法快速制备了TAPB-TFA-COF。其过程是先将两种反应物加入DMSO溶剂中超声5 min,超声下慢慢加入少量(1.6 mL)乙酸溶液;然后将反应液密封室温反应2 h得到TAPB-TFA-COF,该材料具有高比表面积和良好的热、化学稳定性,可作为DSPE吸附剂。Guo等^[50]^以1,3,5-三(4-甲酰基苯基)苯(TFPB)和联苯二胺为单体,通过单体诱导原位室温法在氨基功能化的不锈钢纤维上制备了的TFPB-BD涂层,化学键合的TFPB-BD纤维涂层具有高稳定性和优异的吸附性能,用于SPME水产品中痕量多氯联苯。目前,室温法是制备I-COFs材料的最佳方法之一,且为I-COFs材料的量化生产提供了可能。

综上,I-COFs材料的合成方法灵活多样,这为其高效、快速的制备提供了丰富的选择性。简单温和的制备方法是I-COFs材料实现广泛应用的前提和保障。

## 3 亚胺类COFs材料在样品前处理中的应用

目前,I-COFs材料的合成研究正处于快速发展的阶段,不少性能优异、应用潜力较大的COFs已被合成,这为COFs材料在样品前处理介质中的应用研究提供了丰富的来源和选择。I-COFs的有机单体种类丰富,采用不同结构或功能基团的有机单体可以设计合成不同结构和种类的COFs富集介质。I-COFs材料高比表面积、高孔隙率的性能赋予了它极高的吸附负载量,且可通过*π-π*作用、疏水作用、氢键作用等多重分子间协同作用使目标分析物被高效富集;通过精确调控COFs的孔洞结构和功能化基团,从而实现目标痕量物质的选择性富集。目前,I-COFs材料作为新型萃取介质在固相萃取、磁性固相萃取、分散固相萃取和固相微萃取技术应用中受到了广泛关注。近年来,基于I-COFs材料的SPE、MSPE、DSPE和SPME在样品前处理中的应用情况统计见图2。

**图2:**
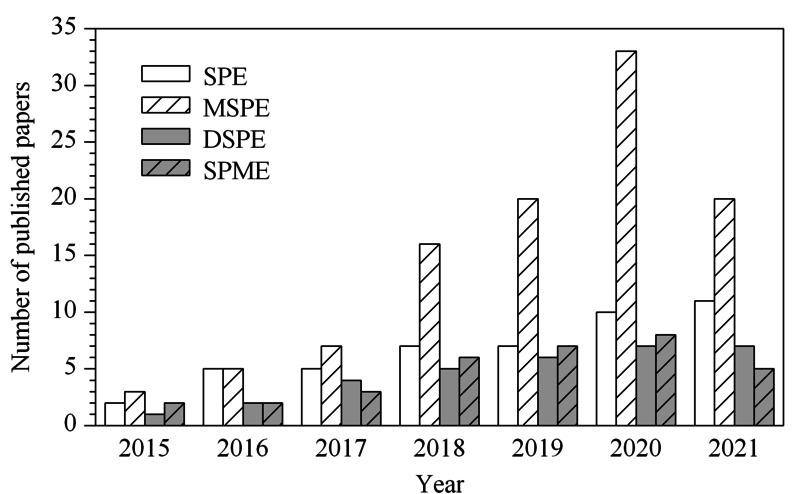
I-COFs材料在SPE、MSPE、DSPE和SPME中应用情况的统计

### 3.1 固相萃取

固相萃取具有操作简便、重现性好、高通透性和高富集倍数等优点,且易于在线、自动化与分析仪器联用,吸附剂是固相萃取技术的关键。I-COFs材料具有高比表面积、高孔隙率和稳定性好等优点,适合用作SPE柱填料。采用1,3,5-三甲酰基间苯三酚与4,4'-偶氮二苯胺、2,6-二氨基蒽醌为有机单体制备的TpAzo和DAAQ-Tp(原文写作DAAQ-TFP)材料,作为SPE吸附剂可通过*π*-*π*作用、疏水作用和氢键作用高效富集苯甲酰脲类化合物,成功用于果蔬样品中的苯甲酰脲类化合物分离分析。结果表明,果汁中检出了0.41 ng/mL氟苯脲,另外黄瓜与番茄中分别检出0.18 ng/g和0.34 ng/g的杀铃脲^[39,40]^。Wang等^[51]^使用COF-LZU1作为SPE吸附剂,结合表面辅助激光解吸电离飞行时间质谱分析检测,能快速高灵敏分析水样中的氟化合物,其检出限可达ng/L或以下水平。引入特定的功能基团是提高COFs材料吸附选择性的重要途径,Ji等^[52]^用乙烯基COF与4-氨基苯硫醇通过“巯-烯”点击反应合成了氨基修饰的NH_2_@COF,由于引入的-NH_2_能与羧酸类农药中的-COOH产生相互作用,与乙烯基COF和商用吸附剂相比,NH_2_@COF SPE吸附剂对羧酸类农药的萃取效率更高,NH_2_@COF-SPE结合HPLC联用分析,在最优条件下方法的线性范围为0.2~100 ng/mL(*r*>0.999), LOD为0.010~0.060 ng/mL,该方法成功应用于环境水样中6种羧酸类农药的分析检测。与其他功能材料复合可以提高COFs材料的化学稳定性和应用能力,Li等^[53]^以通过溶剂热法制备的共价有机骨架功能化聚(苯乙烯-二乙烯基苯-甲基丙烯酸缩水甘油酯)复合材料(COF@PS-GMA)作为注射器内SPE的吸附材料对环境水样中非甾体类抗炎药进行了富集净化,结果表明:COF@PS-GMA材料主要通过*π-π*作用实现目标物的有效富集,结合HPLC联用分析可灵敏、准确地检测废水样品中的7种非甾体类抗炎药。I-COFs材料中具有特定的功能基团是实现目标痕量物质选择性富集的关键因素,功能化I-COFs材料是拓展其应用范围的有效途径。分子印迹聚合物(molecularly imprinted polymer, MIP)是对某一特定的目标分析物具有选择性识别性能的聚合物,将MIP与COFs结合可以构建高选择性和高萃取容量的吸附材料。Ji等^[47]^制备的MICOFs吸附剂对4种结构相似的氰基拟除虫菊酯具有高选择性和高萃取容量,以SPE-HPLC法测定植物样品中氰基拟除虫菊酯,线性范围为0.1~200 ng/g(*r*>0.9981), LOD为0.011~0.018 ng/g,研究表明MICOFs能从复杂基质中选择性高效提取氰基拟除虫菊酯。

在线萃取可以提高分析速度和萃取效率,我们^[54]^以均苯三甲酰肼和对苯二甲醛为有机单体设计合成了一种新的腙键COFs,用于在线微固相萃取苏丹染料,利用COFs骨架强的疏水性、丰富的苯环和亚胺键实现了苏丹染料的高效富集,与3种商用的吸附剂相比,腙键COFs具有较好的萃取性能;研究表明,COFs与目标分析物之间的萃取机理主要基于*π-π*作用、疏水作用和氢键作用等多重协同作用;通过在线萃取结合HPLC联用实现了辣椒粉和香肠样品中苏丹红染料的高灵敏、快速准确分析检测。Liu等^[55]^用联苯二胺与2,4,6-三羧基-1,3,5-三甲酰基苯(CTp)为原料制备了CTpBD COFs,由于COFs的高比表面积以及骨架中的-COOH基团能与金属离子发生配位作用,作为在线SPE吸附剂萃取痕量金属离子,采用流动注射富集与电感耦合等离子体质谱联用分析检测,在最优条件下,方法的线性范围为0.05~25 μg/L, LOD为2.1~21.6 ng/L,最后成功用于水样和食品中多种金属离子的应用分析,CTpBD COFs材料中特异性功能基团(-COOH)是实现金属离子选择性富集的主要因素。选择具有合适粒径和机械稳定性的COFs材料,是发展COFs在线固相萃取技术的关键。

### 3.2 磁性固相萃取

磁性固相萃取具有操作简单、快速、兼容性和选择性好等特点,而I-COFs材料的比表面积大、稳定性好、结构可功能化,是制备磁性复合材料的最佳选择。近来,基于I-COFs材料的磁性萃取介质研制和应用受到高度关注。Li等^[56]^以1,3,5-三甲酰基间苯三酚和联苯二胺为有机单体,采用单体诱导原位生长法制备了核-壳可控的Fe_3_O_4_@TpBD,用于吸附和去除环境水样中的双酚类污染物,由于TpBD具有强的疏水性和丰富的亲水基团,材料对双酚A和双酚AF表现出优异的萃取性能,其最大吸附容量分别达160.6 mg/g和236.7 mg/g。He等^[43]^受满天星花束的启发,研制出新型花束形的磁性Fe_3_O_4_@TpPa-1,由于TpPa-1富含N、O元素和大*π-π*骨架,材料对多环芳烃(PAHs)有较好的富集能力,建立了MSPE HPLC-FLD法分析环境水样中6种PAHs的方法,与其他磁性材料的检测方法相比,该方法的LOD最低。Lin等^[46]^以1,3,5-三(4-氨基苯基)苯与2,5-二羟基对苯二甲醛为单体室温法合成了TAPB-Dha@Fe_3_O_4_(原文献写作COF-DtTb@Fe_3_O_4_),用于萃取牛奶样品中的有机磷农药残留,建立了MSPE LC-MS/MS分析方法,与其他分析方法相比,该方法不需要蛋白质沉淀,且具有更大的富集倍数(50倍)和更低的LOD(0.001~0.01 μg/L)。最近,Li等^[57]^报道了一种基于Zr^4+^与磷酸之间的配位作用来选择性高效富集有机磷农药,他们首先以单分散的Fe_3_O_4_为磁核,1,3,5-三甲酰基间苯三酚、联苯二胺和(4-氨基苯基)磷酸缩合后的COFs为壳层,采用一锅法合成磷酸功能化磁性COF(Fe_3_O_4_@COF-PA);再将Fe_3_O_4_@COF-PA与ZrOCl_2_通过后合成修饰制备了Fe_3_O_4_@COF@Zr^4+^。复合材料通过*π-π*、氢键和配位作用实现了对有机磷农药的选择性高效富集,建立的磁固相萃取-气相色谱-火焰光度检测方法成功地应用于蔬菜样品中7种有机磷农药残留量的测定,方法的检出限低、回收率好。化学共沉淀法是制备磁性纳米复合材料最经济、最简单的方法,Pang等^[58]^以共沉淀法制备了COF-(TpBD)/Fe_3_O_4_,用于分离富集饮料样品中的邻苯二甲酸酯类化合物(PAEs),结合GC-MS/MS分析检测,大大降低了基质效应,所得方法成功用于饮料样品中的15种PAEs的分离分析。MIP对构建高选择性COFs复合材料具有重要作用,Zhao等^[48]^研制的磁性MCMIPs-DAAQ可高效吸附植物样品中的花青素-3-*O*-葡萄糖苷,具有良好的萃取选择性,且吸附剂具有好的稳定性,可重复使用10次。

多肽在复杂生物样品中的分离是蛋白激酶信号转导途径研究的关键一步,选择性富集痕量蛋白质或肽段对基于质谱分析的蛋白质组研究必不可少。I-COFs材料由于物化稳定性好、表面积大、孔结构可调以及便于后修饰等优点,其可能是捕获生物分子潜在的候选材料。Lin等^[44]^将核-壳结构的Fe_3_O_4_@COF复合材料用于富集生物样品中的多肽,同时排阻蛋白质,结果表明:Fe_3_O_4_@COF对芳环数目较多的肽类具有高效富集能力,还可以利用COF的孔径尺寸有效排阻蛋白质大分子。Gao等^[59]^以1,3,5-三甲酰基苯和联苯二胺为单体,在Fe_3_O_4_纳米颗粒表面制备了核-壳型Fe_3_O_4_@Bta-BD(原文献写作Fe_3_O_4_@TbBd)材料,该材料优异的物化性能使其也能选择性富集疏水性多肽同时尺寸排阻蛋白质,结合HPLC-Q-TOF/MS检测,实现了人血清消解液中29个疏水性多肽的高灵敏分离分析。糖基化被认为是蛋白质和多肽最重要的翻译修饰之一,与细胞代谢过程和多种生物学过程的调节密切相关。发展高选择性的吸附剂和高效的样品预处理方法对蛋白质的糖基化分析尤显关键。Wang等^[60]^采用两步溶剂热法制备了Fe_3_O_4_@TpPa-1,用于富集亲水性的糖肽,所发展的MSPE方法可以从IgG酶解液中检测到37个糖肽,LOD低至28 fmol;从人体血清酶解液中检测出114个糖蛋白和228个糖肽,与商用亲水相互作用色谱材料相比,Fe_3_O_4_@TpPa-1具有更高的富集性能。上述研究揭示I-COFs材料在蛋白质组学领域具有良好的应用前景。海洋生物毒素为海洋生物体内存在的一类高活性的特殊代谢成分,是海洋药物的研究热点。Romero等^[61]^用多巴胺原位功能化快速得到氨基修饰的磁性纳米颗粒,再分别与1,3,5-三甲酰基间苯三酚和3,3-二甲基联苯二胺单体反应,最后通过原位生长法得到磁性mTpBD-Me_2_复合材料,用于MSPE吸附剂提取海洋生物毒素,对冈田软海绵酸(OA)和鳍藻毒素-1(DTX-1)的最大吸附容量分别为812 mg/g和830 mg/g,与常用无磁性大孔树脂的吸附性能相比,OA和DTX-1的吸附容量分别增加了约500倍和300倍。结果表明,COFs材料可以高效吸附生物毒素,在环境监测方面具有良好的应用潜力。

### 3.3 分散固相萃取

分散固相萃取是将吸附剂直接分散于液体样品中,这样极大地增加了吸附剂与基质之间的接触面积,可以使目标分析物与吸附剂迅速达到吸附平衡,从而减少萃取时间实现高效富集。分散固相萃取集萃取和净化于一体,操作简便、高效快捷、溶剂用量少。I-COFs材料独特的物理化学性能使其可以作为一种理想的分散固相萃取吸附剂。

利用硫(S)和金属的亲和作用,设计含有丰富硫元素的I-COFs材料可去除水样中有毒金属离子。Ding等^[62]^采用“自下而上”策略合成了一种硫醚功能化的腙键COF-LZU8,其大的*π*共轭结构作为荧光信号传感器,均匀密集的硫醚基团作为Hg^2+^受体,有序的孔道结构有利于金属离子的快速传质和检测,结合腙键的高稳定性,COF-LZU8可同时实现选择性传感检测和去除水样中的Hg^2+^。Sun等^[63]^采用后合成修饰法制备了具有明确介孔结构的共价有机骨架,他们首先选择2,5-二乙烯基对苯二甲醛和1,3,5-三(4-氨基苯基)苯为原料合成乙烯基官能化的骨架材料COF-V,再用偶氮二异丁腈引发COF-V与1,2-乙二硫醇间的“巯-烯”点击反应得到硫基螯合的COF-S-SH。Hg^2+^吸附研究表明,COF-S-SH对Hg^2+^具有非常强的亲和力,吸附容量达1350 mg/g。更重要的是,COF-S-SH具有超高的分配系数值(*K*_d_=2.3×10^9^ mL/g),可以快速有效地将Hg^2+^浓度从5 mg/L降低到0.1 μg/L,该浓度远低于饮用水中可接受的限值(2 μg/L)。这种优异的汞吸收能力可归因于COF-S-SH孔道表面密集分布的硫基官能团的高度亲和力与有序介孔的快速传质,同时也揭示了COFs作为环境修复吸附材料的巨大应用潜力。Xiong等^[64]^提出了一种新型的吸附概念,首先以1,3,5-三甲酰基间苯三酚和2-磺酸对苯二胺为单体制备出COF-SO_3_H,然后用氨水对COF-SO_3_H进行氨化得到[NH_4_^+^][COF-SO_3_^-^]多孔吸附剂,用于铀的吸附研究,结果表明:未氨化COF-SO_3_H的铀吸附容量为360 mg/g,而[NH_4_^+^][COF-SO_3_^-^]的铀吸收量达851 mg/g,氨化吸附剂显著提高了铀的吸附性能。该吸附材料基于离子交换和配位作用协同吸附放射性铀离子,具有优异的化学稳定性和选择性以及良好的循环使用率。上述研究表明,在I-COFs材料中引入特定的功能基团是实现金属离子选择性富集的重要因素,同时揭示I-COFs材料在金属离子分离富集方面具有良好的发展前景。Gao等^[49]^以在室温下制备的TAPB-TFA-COF作为DSPE吸附材料用于硝基芳族化合物的分离富集。TAPB-TFA-COF结构中大量的苯环和F原子能与目标分析物之间形成较强的*π-π*作用、氢键作用和F-F作用,建立了分散固相萃取-高效液相色谱-二极管阵列检测器联用分析水样中6种硝基芳族化合物,方法的检出限低、精密度高。最近,Garai等^[20]^制备的两种COFs(CX4-BD-1和CX4-BD-2)骨架中杯芳烃单元具有很高的表面负电荷,在电荷选择性染料去除方面展现出优异的性能,无论其分子大小如何,均对阳离子染料具有出色的选择性。

核酸适配体是一种可以特异性地识别目标物的寡聚核苷酸,能与相应的配体进行高亲和力和强特异性的结合,其COF复合材料具有很好的应用前景。Ge等^[65]^报道了一种适配体修饰金纳米颗粒掺杂的共价有机骨架复合材料(IBAs-AuNPs/COF),先用2,4,6-三(4-氨基苯基)-1,3,5-三嗪和2,5-二甲氧基对苯二甲醛为有机单体构建COF,再将金纳米颗粒(AuNPs)固定在聚多巴胺修饰的COF上,最后通过Au-S键将含有巯基的胰岛素适配体(IBAs)连接在AuNPs的表面,得到IBAs-AuNPs/COF。由于COF优异的吸附性能以及胰岛素与适配体之间特异性的识别能力,在50倍干扰物质(人免疫球蛋白、溶菌酶和生物素)存在的条件下,IBAs-AuNPs/COF材料也能选择性高效富集人血清样品中的胰岛素。适配体功能化COF是拓展I-COFs材料选择性应用的重要途径。蛋白质磷酸化在细胞信号传递、增殖和分化等多种生物学过程中起着关键作用。然而,由于生物样品中磷酸肽含量低、基质干扰严重,磷酸肽无法直接有效地进行MS测定。因此,在MS鉴定前使用高效的磷酸肽富集和分离方法是十分有必要的。I-COFs材料因其优异的物化性能,有望作为捕获生物分子的良好介质。Wang等^[66]^根据固定金属离子亲和色谱法(IMAC)原理,利用Ti^4+^和酮-烯胺基团间的配位反应将Ti^4+^直接固定在TpPa-2 COFs骨架中,得到一种基于COF的IMAC复合材料(TpPa-2-Ti^4+^)。该材料用于磷酸化肽的富集,因负载了高密度的金属离子和良好有序的孔道结构而具有优异的质量传递和富集性能,可以对实际样品(酪蛋白、Hela细胞、脱脂牛奶)中的磷酸化肽进行高灵敏度和选择性富集。糖肽来源于糖蛋白,具有丰富的亲水功能基。基于相似相溶原理,可以将生物样品中的亲水性糖肽提取到亲水性COFs上。Ma等^[67]^以TpPa-1作为一种亲水性的吸附材料富集IgG酶解液中的*N*-糖肽,结果表明,TpPa-1对*N*-糖肽具有非常高的选择性,甚至在非*N*-糖肽浓度为1000倍的条件下也能实现*N*-糖肽的选择性富集,其灵敏度可达到fmol水平。同时,TpPa-1还具有良好的稳定性、可重复使用性和较高的吸附容量。研究表明,COFs材料在复杂生物样品中具有良好的应用潜力。

### 3.4 固相微萃取

固相微萃取是一种集采样、萃取、浓缩和进样于一体的样品前处理技术,具有操作简单、方便快捷、高效灵敏、环境友好、易于在线和自动化分析等优点。涂层是SPME技术的核心,是影响分析方法灵敏度和选择性的关键因素。近来,COFs SPME涂层成为新型萃取介质的研究热点。Zhang等^[68]^采用物理涂覆法在功能化不锈钢丝上制备了COF-SCU1 SPME涂层,结合GC-MS成功用于室内空气中挥发性苯系物的分离分析。Guo等^[69]^报道以1,3,5-三(4-醛基苯氧基)苯与对苯二甲酰肼为单体,4-羟基苯甲酰肼为调节剂,在溶剂热条件下合成了OH-TPB-COFs,用于SPME涂层萃取富集PAEs。调节剂的引入增强了OH-TPB-COFs的结晶性,当调节剂含量为50%时,OH-TPB-COFs具有最好的晶体结构;同时归于涂层与分析物之间的*π-π*作用、氢键作用和疏水作用,显著提高了对PAEs的萃取效率,建立了SPME-GC-FID法测定水样中PAEs的方法,方法的线性范围宽、回收率和重现性好。

物理涂覆法是制备SPME涂层最简便的方法之一,但由于涂层与基底的物理黏附力较弱,涂层的稳定性和重现性较差,所以采用化学键合法是增强涂层稳定性和重现性的重要途径。Wu等^[70]^以1,3,5-三甲酰基苯和对苯二甲酰肼为有机单体,在多巴胺修饰的不锈钢丝表面制备了腙键结构的COF涂层,与气相色谱-电子捕获检测器联用分析拟除虫菊酯类农药残留。基于COF与目标分析物之间的疏水作用、*π-π*作用和氢键作用,COF涂层具有出色的萃取性能,萃取容量优于商用涂层,所发展的方法实现了蔬菜和水果样品中拟除虫菊酯类农药的高灵敏分离分析。随后,他们^[71]^以均苯三甲酰肼和4-羟基间苯二甲醛为原料,采用“巯-烯”点击反应制备的腙键COF涂层对有机氯农药也展现出优异的富集能力,富集因子高达2190~10998。Ma等^[72]^以聚多巴胺为交联剂,在不锈钢丝上制备了TpBD SPME涂层,结合GC-MS/MS分析烤肉样品中的16种PAHs, TpBD涂层显示出优异的萃取效率,富集因子达1069~10879, LOD为0.02~1.66 ng/L,且TpBD涂层的萃取性能稳定,单根纤维至少可重复使用200次。Guo等^[50]^以TFPB-BD COF为SPME涂层,建立了SPME-GC-MS/MS测定水产品中痕量多氯联苯的分析方法,方法的富集因子高和检出限低。最近,Pang等^[73]^报道了一种电增强的不锈钢丝SPME COF涂层,他们以2, 6-二氨基蒽醌和1,3,5-三甲酰基间苯三酚为原料,在不锈钢丝表面原位键合制备了导电的DAAQ-Tp(原文献写作DQTP)涂层,用于萃取双酚A。通过在不锈钢丝纤维上外加电场以提高DAAQ-Tp涂层的萃取能力,用电增强的固相微萃取(EE-SPME)DAAQ-Tp纤维与常规的DAAQ-Tp纤维相比,EE-SPME纤维涂层具有更高的萃取效率和更快的萃取时间(10 min内即可达到萃取平衡)。以EE-SPME为萃取装置,结合GC-FID实现了食品包装袋中双酚A的定量测定,结果比较满意。用电增强的萃取技术弥补了常规固相微萃取技术的不足,有利于提高分析方法的灵敏度,拓展COFs材料的应用范围。在线萃取技术可以大大缩短样品分析时间、增加样品分析通量、减少操作程序和人为误差,实现自动化分析的同时提高分析结果的灵敏度及精密度。我们^[74]^采用均苯三甲酰肼和联苯二甲醛为有机单体,通过化学反应原位聚合研制了COFs聚合物整体柱,结合HPLC在线分析磺胺类药物和荧光白试剂,COFs材料表现出优异的富集能力,在线方法实现了水产品中磺胺类药物残留和食品包装袋中荧光白试剂的快速、高灵敏分析检测。研制化学稳定性好且适合低柱压、高通量采样的COFs材料是发展I-COFs材料在线微萃取技术的重要方向。表1总结了近年来I-COFs材料在SPE、MSPE、DSPE和SPME中的代表性应用实例。

**表 1:** I-COFs材料在SPE、MSPE、DSPE和SPME中的代表性应用实例

Extraction technology	I-COFs	Detection technology	Sample matrix		Analytical substance		Enrichment factors		Repeated times		LODs	Ref.
SPE	DAAQ-Tp	HPLC-UV	environmental water, food		BUs		-		30		0.02-0.05 ng/mL, 0.02-0.08 ng/g	[40]
	MICOFs	HPLC-DAD	plants		cyano pyrethroids		-		50		0.011-0.018 ng/g	[47]
	NH_2_@COF	HPLC-DAD		environmental water		carboxylic acid pesticides		-		50	0.010-0.060 ng/mL	[52]
	COF@PS-GMA	UHPLC-UV		wastewater		NSAIDs		-		20	0.13-0.82 μg/L	[53]
	HL-COP	HPLC-UV		food		sudan dyes		305-757		-	0.03-0.15 μg/L	[54]
	CTpBD	ICP-MS		environmental water, food		metal ions		10		20	2.1-21.6 ng/L	[55]
MSPE	Fe_3_O_4_@TpPa-1	HPLC-FLD		environmental samples		PAHs		-		-	0.24-1.01 ng/L	[43]
	Fe_3_O_4_@COF	HPLC-Q-TOF/MS		human serum		peptides		-		10	-	[44]
	TAPB-Dha@Fe_3_O_4_	LC-MS/MS		fatty milk		OPPs		50		6	0.001-0.010 μg/L	[46]
	Fe_3_O_4_@COF@Zr^4+^	GC-FPD		vegetables		OPPs		-		6	0.7-3.0 μg/kg	[57]
	COF-(TpBD)/Fe_3_O_4_	GC-MS/MS		beverage		PAEs		-		4	0.005-2.748 μg/L	[58]
	Fe_3_O_4_@TpPa-1	Nano LC-MS/MS		human serum digests		N-glycopeptides		-		5	28 fmol	[60]
DSPE	CX4-BD-1, CX4-BD-2	UV-vis		environmental water		cationic dyes		-		-	-	[20]
	TAPB-TFA-COF	HPLC-DAD		environmental water		NACs		-		9	0.03-0.09 mg/L	[49]
	COF-LZU8	ICP-OES		water		Hg^2+^		-		3	25.0 μg/L	[62]
	[N] [COF-S]	XPS		seawater		uranium ions		-		6	-	[64]
	IBAs-AuNPs/COF	MALDI-TOF-MS		human serum		insulin		-		-	0.28 μg/L	[65]
	TpPa-2-Ti^4+^	LC-MS/MS		tryptic digests		phosphopeptides		-		-	4 fmol	[66]
SPME	TFPB-BD	GC-MS		aquatic products		PCBs		4471-7488		180	0.07-0.35 ng/L	[50]
	COF-SCU1	GC-MS/MS		indoor air		benzene homo- logues		276-887		100	0.03-0.15 ng/L	[68]
	OH-TPB-COF	GC-FID		environmental water		PAEs		-		60	0.032-0.451 μg/L	[69]
	Hydrazone COF	GC-ECD		vegetables, fruits		pyrethroids		307-2327		150	0.11-0.23 μg/kg	[70]
	TpBD	GC-MS/MS		grilled meat		PAHs		1069-10879		200	0.02-1.66 ng/L	[72]
	DAAQ-Tp	GC-FID		food packagings		BPA		-		-	3 μg/L	[73]

MSPE: magnetic solid phase extraction; DSPE: dispersive solid phase extraction; vis: visible spectrophotometer; DAD: diode array detector; UHPLC: ultra-high performance liquid chromatography; ICP: inductive coupled plasma; ECD: electron capture detector; FPD: flame photometric detector; FLD: fluorescence detector; XPS: X-ray photoelectron spectroscopy; OES: optical emission spectrometer; BUs: benzoylurea insecticides; NSAIDs: non-steroidal anti-inflammatory drugs; PAHs: polycyclic aromatic hydrocarbons; OPPs: organophosphorus pesticide; PAEs: phthalate esters; NACs: nitroaromatic compounds; PCBs: polychlorinated biphenyls; BPA: bisphenol A; “-”indicates data are not available from the corresponding reference.

近年来,基于I-COFs材料诸多优异的物理化学特性,其结合SPE、MSPE、DSPE和SPME实现了各种复杂基质样品(食品、环境、植物、生物等)中不同目标分析物(农药兽药残留、食品添加剂、重金属离子、环境污染物、肽类和生物毒素等)的有效分离富集,其应用范围广、分析物种类多,同时也揭示了I-COFs材料良好的应用潜力。

## 4 结论与展望

综上,I-COFs材料作为新型萃取介质在样品前处理领域已展现出良好的应用前景。然而,相对于结构种类繁多的I-COFs材料而言,目前仅有少数I-COFs用于样品处理领域的应用研究,更多极具应用潜力的I-COFs材料还有待研究人员去发掘。从目标分析物来看,大多数分析物都具有苯环结构,萃取机理主要基于疏水、*π-π*和氢键等作用协同实现目标分析物分离富集,I-COFs材料的吸附选择性还有待提高。在萃取技术方面,现有I-COFs材料的萃取技术较少,发展I-COFs材料与多种萃取方式相结合,更能突显其在样品前处理方面的优势。目前,I-COFs材料在样品处理领域的应用研究还处于发展阶段,今后可从以下四方面开展相关研究:(1)设计开发更多性能优异的I-COFs材料(3D、手性、超高比表面积)应用于样品前处理领域,有望解决一些传统材料难以解决的分离富集问题;(2)引入特异性功能基团或结合选择性材料(分子印迹、适配体、免疫吸附剂),利用特殊的分子间作用力或主-客体适配原则等提高I-COFs材料的吸附选择性;(3)发展基于I-COFs的新型萃取介质(管状、管内涂层、整体柱),或与其他功能材料复合,拓展I-COFs材料在样品处理领域的应用能力和范围;(4)发展I-COFs材料的新型萃取技术(在线、集成化、自动化)。

总之,随着研究人员对I-COFs材料的不断探索和认识,必将会有更多性能优异的I-COFs应用于样品前处理领域,样品种类和分析对象会更加广泛、应用前景会更加广阔。
